# Total Mechanical Unloading Minimizes Metabolic Demand of Left Ventricle and Dramatically Reduces Infarct Size in Myocardial Infarction

**DOI:** 10.1371/journal.pone.0152911

**Published:** 2016-04-28

**Authors:** Keita Saku, Takamori Kakino, Takahiro Arimura, Takafumi Sakamoto, Takuya Nishikawa, Kazuo Sakamoto, Masataka Ikeda, Takuya Kishi, Tomomi Ide, Kenji Sunagawa

**Affiliations:** 1 Department of Therapeutic Regulation of Cardiovascular Homeostasis, Center for Disruptive Cardiovascular Medicine, Kyushu University, Fukuoka, Japan; 2 Department of Cardiovascular Medicine, Graduate School of Medical Sciences, Kyushu University, Fukuoka, Japan; 3 Collaborative Research Institute of Innovative Therapeutics for Cardiovascular Diseases, Center for Disruptive Cardiovascular Medicine, Kyushu University, Fukuoka, Japan; University of Pecs Medical School, HUNGARY

## Abstract

**Background:**

Left ventricular assist device (LVAD) mechanically unloads the left ventricle (LV). Theoretical analysis indicates that partial LVAD support (p-LVAD), where LV remains ejecting, reduces LV preload while increases afterload resulting from the elevation of total cardiac output and mean aortic pressure, and consequently does not markedly decrease myocardial oxygen consumption (MVO_2_). In contrast, total LVAD support (t-LVAD), where LV no longer ejects, markedly decreases LV preload volume and afterload pressure, thereby strikingly reduces MVO_2_. Since an imbalance in oxygen supply and demand is the fundamental pathophysiology of myocardial infarction (MI), we hypothesized that t-LVAD minimizes MVO_2_ and reduces infarct size in MI. The purpose of this study was to evaluate the differential impact of the support level of LVAD on MVO_2_ and infarct size in a canine model of ischemia-reperfusion.

**Methods:**

In 5 normal mongrel dogs, we examined the impact of LVAD on MVO_2_ at 3 support levels: Control (no LVAD support), p-LVAD and t-LVAD. In another 16 dogs, ischemia was induced by occluding major branches of the left anterior descending coronary artery (90 min) followed by reperfusion (300 min). We activated LVAD from the beginning of ischemia until 300 min of reperfusion, and compared the infarct size among 3 different levels of LVAD support.

**Results:**

t-LVAD markedly reduced MVO_2_ (% reduction against Control: -56 ± 9%, *p*<0.01) whereas p-LVAD did less (-21 ± 14%, *p*<0.05). t-LVAD markedly reduced infarct size compared to p-LVAD (infarct area/area at risk: Control; 41.8 ± 6.4, p-LVAD; 29.1 ± 5.6 and t-LVAD; 5.0 ± 3.1%, *p*<0.01). Changes in creatine kinase-MB paralleled those in infarct size.

**Conclusions:**

Total LVAD support that minimizes metabolic demand maximizes the benefit of LVAD in the treatment of acute myocardial infarction.

## Introduction

Although early revascularization therapy for acute myocardial infarction (MI) has reduced acute-phase mortality markedly to less than 10% [1, 2], 20–30% of acute MI patients ultimately develop heart failure [3]. The mortality in patients with heart failure remains high despite recent advances in drug and/or device therapy [4]. The amount of myocardial damage in the early phase of MI governs the progression to heart failure [5]. The current approach of reperfusion therapy does not adequately prevent future heart failure. Thus there is clearly an unmet need for better strategy in the treatment of acute MI.

Over the past few decades, significant progress has been made in the development of left ventricular assist device (LVAD). In particular, implantable LVADs have been shown to improve the long-term survival and quality of life in patients with severely compromised heart failure [6]. Furthermore, the recent development of transvascular LVADs such as TandemHeart^®^ (Cardiac Assist Inc., Pittsburgh, Pa) and Impella^®^ (Abiomed, Inc., Danvers, MA) have revolutionized both the treatment of cardiogenic shock [7, 8] and high-risk percutaneous coronary intervention [9].

**Fig 1:**
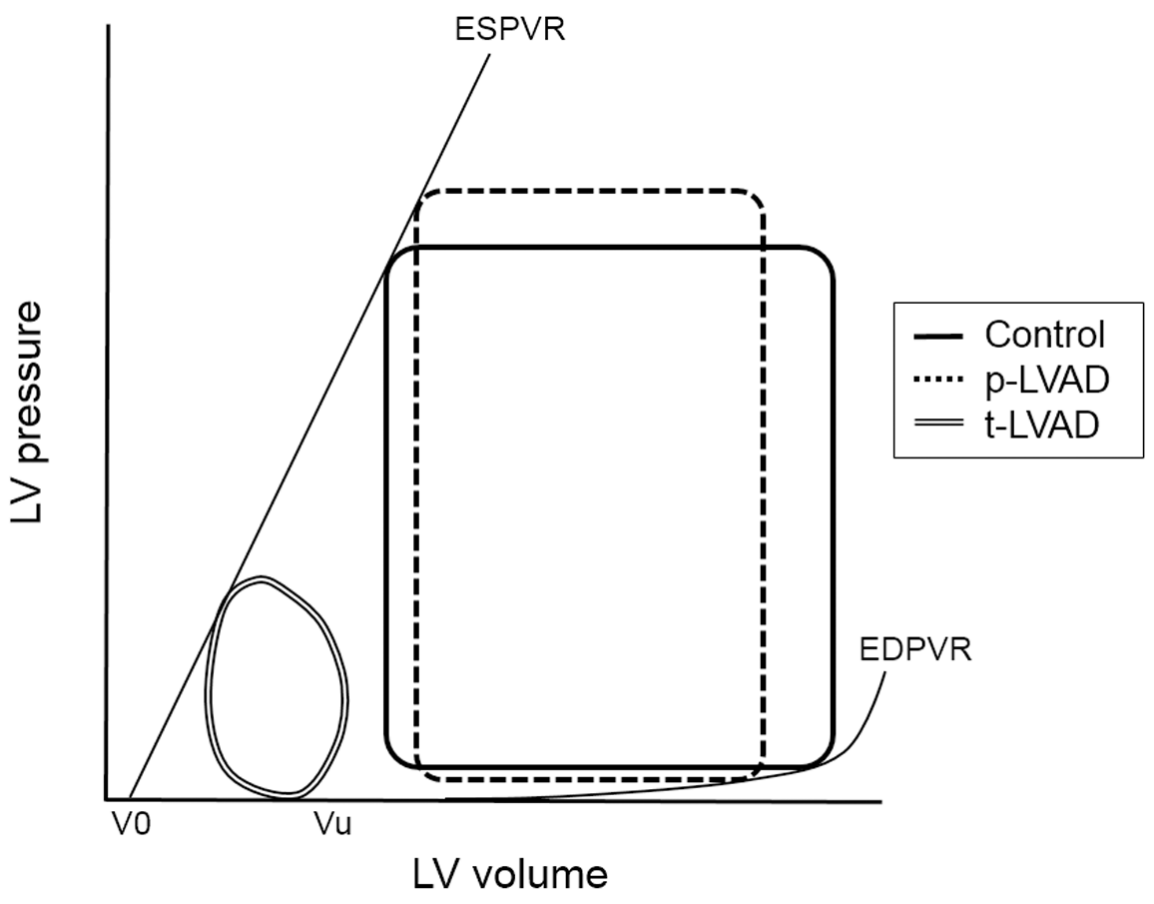
Theoretical analysis of the impact of left ventricular assist device (LVAD) on pressure-volume loop in a normal left ventricle. The solid line represents the baseline pressure-volume (PV) loop. In partial LVAD support (p-LVAD), LVAD decreases left ventricle (LV) end-diastolic volume and increases mean arterial pressure (AP), which in turn increases end-systolic volume (dotted line). On the other hand, total LVAD support (t-LVAD) markedly lowers LV pressure to below AP yielding an extremely small PV area (double line). EDPVR, end-diastolic pressure volume relation; ESPVR, end-systolic pressure volume relation;.

The pressure-volume area (PVA) is a specific area circumscribed by the end-systolic and end-diastolic pressure-volume curves and the systolic segment of the pressure-volume trajectory in contraction. The PVA represents total mechanical energy which consists of external work (EW) and potential energy (PE), and correlates linearly with myocardial oxygen consumption (MVO_2_) [10]. In this framework, LVAD decreases preload by withdrawing blood from the left ventricle (LV) and increases afterload by infusing blood into the aorta to augment net cardiac output. Therefore, the impact of LVAD on PVA depends on the balance between the decreased preload and increased afterload. In partial LVAD support (p-LVAD), in which the LV continues to eject, the LVAD decreases LV end-diastolic volume and increases mean arterial pressure (AP), which in turn increases end-systolic volume. Thus, p-LVAD does not reduce PVA markedly. On the other hand, in total LVAD support (t-LVAD), the LV no longer ejects because LV pressure is lower than the AP due to low LV preload. t-LVAD renders PVA extremely small (Fig 1). Therefore, theoretical analysis indicates that the decreasing in PVA and MVO_2_ of LV is much greater in t-LVAD than p-LVAD.

On the basis of these backgrounds, we focused on the mechanical impact of LVAD on LV, and hypothesized that t-LVAD during acute phase of MI minimizes MVO_2_ and reduces infarct size, because an imbalance between oxygen supply and demand is the fundamental underlying pathophysiology of MI. The purpose of this study was to evaluate the differential impact of the support level of LVAD on PVA, MVO_2_ and infarct size in a canine model of ischemia-reperfusion.

## Materials and Methods

### Animals and surgical preparations

Experiments and animal care were approved by the Committee on Ethics of Animal Experiments, Kyushu University Graduate School of Medical Sciences, and performed strictly in accordance with the Guide for the Care and Use of Laboratory Animals published by the US National Institutes of Health.

Twenty-three mongrel dogs (16.3 ± 5.0 kg) purchased from KBT Oriental, Co., Ltd. (Saga, Japan) were used in this study. Each dog was housed in an individual cage measuring 1305 cm in height, 850 cm in width, and 1000 cm in length (WORKTEC Co., Ltd., Ishikawa, Japan) in a room maintained at a temperature of 20°C and a 12-h light/dark cycle. The dogs were acclimatized to the laboratory environment for at least 7 days prior to the experiments. Since the experiments were completed within one day, the dogs were kept in the cages for a total of 8–14 days. The dogs were fed a commercial dog food (PC-2, Oriental Yeast, Co., Ltd.) once per day in the morning and had free access to tap water. All the animals received regular contact, at least daily, with the animal attendant and/or researchers for socialization and to ensure that they were adapted to the laboratory environment. Anesthesia was initiated by intravenous pentobarbital (25 mg/kg) and pancuronium bromide (0.08 mg/kg). The dog was intubated and artificially ventilated with room air to maintain physiological pH and oxygen saturation. Body temperature was maintained between 37 and 38°C. Continuous isoflurane (1–2%) inhalation was used to maintain an appropriate level of anesthesia during the experiment. A catheter (6-Fr) was placed in the left femoral vein for administering fluids. We measured systemic AP, right atrial pressure (RAP) and left atrial pressure (LAP) by fluid-filled catheter-transducer systems (Model DX-200; Nihon Kohden, Tokyo, Japan). After a median sternotomy, a pericardial incision was made and an ultrasonic flow probe (Model 12PSB 508; Transonic, Ithaca, NY) was placed on the ascending aorta to measure cardiac output (CO). In Protocol 1, we also measured flow in the left anterior descending coronary artery (LAD) with an ultrasonic flow probe (Model 1PRB4112; Transonic, Ithaca, NY). To open the baroreflex feedback loop, we isolated the arterial baroreceptors vascularly according to previously reported methods [11]. Briefly, we exposed bilateral carotid arteries and vago-sympathetic trunks through a midline cervical incision. After ligation of bilateral internal and external carotid sinuses, both carotid sinuses were cannulated and connected to a servo-controlled piston pump (Model ET-126A; Labworks Inc., Tokyo, Japan) to maintain carotid sinus pressure (CSP) at a constant level. A 5-Fr catheter was inserted into the coronary sinus via the right jugular vein for blood gas sampling. In Protocol 2, we measured LV pressure using a 5-Fr high-fidelity micromanometer (Model PC-751; Millar Instruments, Houston, TX) in the t-LVAD group. Total experimental duration was 3–3.5 h in protocol 1 and 7.5–8 hours in protocol 2.

### Left ventricular assist device

**Fig 2:**
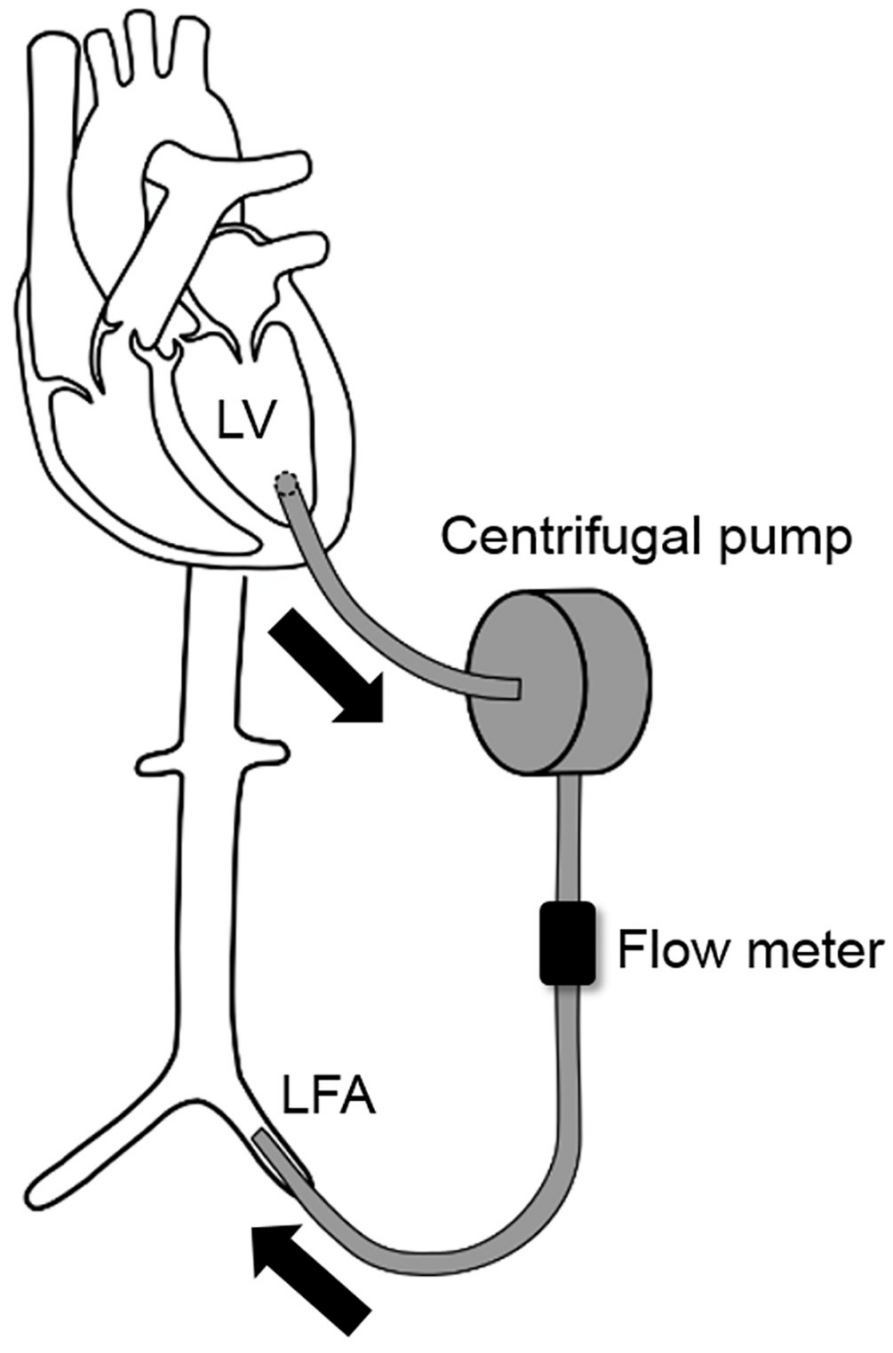
Diagram of the left ventricular assist device (LVAD) used in this experiment. The inlet and outlet cannulas were inserted into the LV apex and left femoral artery (LFA), respectively. These cannulas were connected to a centrifugal pump and an in-line ultrasonic flow probe was placed in the LVAD circuit to measure LVAD flow continuously.

We constructed a continuous-flow LVAD system by withdrawing blood directly from the LV and pumping into a systemic artery. We inserted the inlet cannula (polyethylene tube, 6 mm internal diameter) into the LV apex and the outlet cannula into the left femoral artery, and then connected these cannulas to a centrifugal pump (CBBPX-80; Medtronic, Minneapolis, MN). An in-line ultrasonic flow probe (Model XL; Transonic, Ithaca, NY) was placed in the LVAD circuit to measure LVAD flow continuously (Fig 2).

### Pressure-volume relationship

We inserted a catheter-tipped micromanometer (Model PC-751; Millar Instruments, Houston, TX) into the LV via the left carotid artery. Two pairs of ultrasonic crystals were implanted in the endocardium of the LV to measure the anterior-posterior (short axis, D_SA_) and base-apex (long axis, D_LA_) dimensions. We calculated the left ventricular volume (LVV) by the modified ellipsoid formula: LVV = (π/6) ×D_SA_ ×D_SA_ ×D_LA_. We occluded the inferior vena cava and determined the end-systolic pressure-volume relationship (ESPVR) including the volume axis intercept V_0_, as well as the end-diastolic pressure-volume relationship (EDPVR). We calculated PVA from the area circumscribed by the ESPVR and EDPVR and the systolic segment of the pressure-volume trajectory in contraction.

### Myocardial oxygen consumption

To determine myocardial oxygen consumption, we collected aortic and coronary sinus blood samples into heparinized syringes. The hemoglobin (Hb) concentration, oxygen partial pressure (PO_2_), and oxygen saturation (SO_2_) were analyzed immediately using an automatic blood gas analyzer (ABL System 625; Radiometer Medical A/S, Copenhagen, Denmark). We estimated MVO_2_ as a product of coronary blood flow (ml/min) and the difference in oxygen content between the arterial (aO_2_) and coronary sinuses (vO_2_), and expressed in ml/min. Oxygen content in blood was expressed in ml O_2_/dl of blood: aO_2_ (vO_2_) = (1.34×Hb×sO_2_/100%) + (pO_2_×0.0031) [12].

### Infarct size assessment

At the conclusion of the experiment in protocol 2, we arrested the heart by injecting potassium chloride. We ligated the LAD at the site where we created MI. We extracted the heart and cannulated the LAD, left circumflex artery, and right coronary artery with 4-Fr tubes. We perfused coronary arteries with 100 ml saline followed by 20 ml Evans’ blue dye at a perfusion pressure of 100 mmHg. We cut the heart into 4 slices perpendicular to the long axis, and placed in a bath of 1% triphenyltetrazolium chloride (TTC) at 37°C for 15 min. We photographed each slice and assessed both the at risk area and the infarct area by tracing each region [13]. We also assessed the level of creatine kinase (CK-MB), a cardiac biomarker, at 300 min after reperfusion.

### Protocols

#### Protocol 1: The impact of LVAD on PVA and MVO_2_

In 5 normal dogs, we changed LVAD flow at 3 levels: Control (no LVAD support), p-LVAD (LVAD flow equals CO) and t-LVAD (total LVAD-dependent circulation), and simultaneously recorded the pressure-volume (PV) loop, PVA and MVO_2_.

#### Protocol 2: The impact of LVAD flow on infarct size in myocardial infarction

We randomly allocated 18 dogs into 3 groups: Control (no LVAD support, n = 8), p-LVAD (n = 5) and t-LVAD (n = 5). We ligated the LAD at the level just above the diagonal branch for 90 min and then reperfused for 300 min. To assess the maximum effect of LVAD support, we started LVAD at the beginning of ischemia and continued LVAD support until the end of the experiment. We compared the area of infarction and CK-MB among the three groups. Hemodynamics including heart rate (HR), AP, CO, LVAD flow, RAP and LAP were recorded simultaneously. In the t-LVAD group, we also measured LV pressure to confirm dissociation of LV pressure from AP and verify whether t-LVAD achieved total LVAD-dependent circulation.

### Data analysis

We digitized time-series data at 200 Hz with a 16-bit analog-to-digital converter (PowerLab 16/35; ADInstruments, USA) and stored on a dedicated laboratory computer system. Data are presented as means ± SD. The JMP software version 11 (SAS institute Inc., NC, USA) was used in statistical analyses. Differences were considered significant when *p* < 0.05. In Protocol 1, the effects of LVAD support on hemodynamics, PVA and MVO2 were evaluated by either paired Student’s t-test or one-way repeated measures ANOVA with Bonferroni post-hoc test. In Protocol 2, hemodynamics, infarct size and CK-MB were evaluated by one-way factorial ANOVA. Tukey-Kramer test was used for post-hoc comparisons.

## Results

### The impact of LVAD support on PVA and MVO_2_

Table 1 shows the hemodynamics and oxygen saturation in coronary sinus at various levels of LVAD support in normal dogs. As LVAD flow increased, mean AP and total cardiac output (CO + LVAD flow) increased slightly but significantly, while LAP decreased significantly. t-LVAD markedly lowered peak LV pressure to below the mean AP, indicating that LV no longer ejected and circulation totally depended on LVAD. Notably, t-LVAD markedly increased the oxygen saturation in coronary sinus (S_CS_O_2_) indicating reduced LV oxygen consumption.

**Table 1:** Impact of level of left ventricular assist device (LVAD) support on hemodynamics in normal dogs.

	HR	CO	LVAD flow	CBF	MAP	LVEDP	Peak LVP	RAP	LAP	ESV	EDV	S_CS_O_2_
	(bpm)	(l/min)	(l/min)	(ml/min)	(mmHg)	(mmHg)	(mmHg)	(mmHg)	(mmHg)	(ml)	(ml)	(%)
Control	119±12	1.19±0.27	0	14.3±2.5	98±12	5.5±2.1	111±9	3.52±2.36	8.25±1.62	25.5±7.1	37.4±5.4	41.9±5.4
p-LVAD	122±9.1	0.58±0.12*	0.71±0.13*	13.3±3.1	105±10*	4.2±1.9*	118±20	3.63±2.29	7.26±2.00	25.0±7.0	33.8±6.3*	49.9±8.7
t-LVAD	122±7.8	0*†	1.46±0.30*†	12.4±5.5	110±7*†	1.2±1.0*†	30±19*†	3.67±2.02	3.63±1.78*†	22.0±5.8*†	27.1±6.2*†	67.7±7.1*†

Data are expressed as means ± SD. Control, no LVAD support; p-LVAD, partial LVAD support; t-LVAD, total LVAD support; HR, heart rate; CO, cardiac output; LVAD flow, flow of left ventricular assist device; CBF, coronary (LAD) blood flow; MAP, mean arterial pressure; LVEDP, left ventricular end-diastolic pressure; Peak LVP, peak LV pressure; RAP, right atrial pressure; LAP, left atrial pressure; ESV, end systolic volume; EDV, end diastolic volume; S_CS_O_2_, oxygen saturation in coronary sinus vein.

* *p* < 0.05 versus Baseline;

^†^ *p* < 0.05 versus p-LVAD.

**Fig 3:**
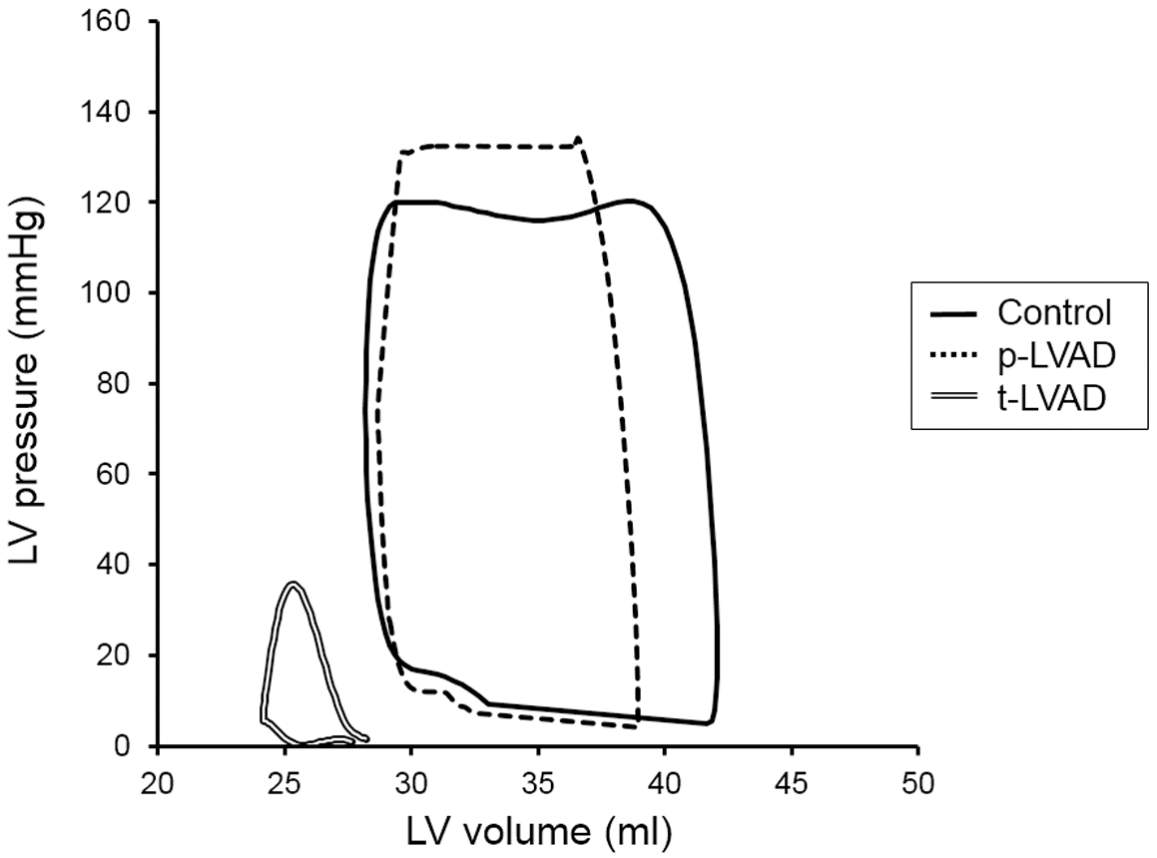
Representative pressure-volume loops at 3 levels of left ventricular assist device (LVAD) support in a normal dog. The solid line represents pressure-volume (PV) loop with no LVAD support (Control). Partial LVAD support (p-LVAD) decreases left ventricle (LV) end-diastolic volume and increases mean arterial pressure (AP), thus PVA does not decrease much (dotted line). On the other hand, total LVAD support (t-LVAD) lowers LV pressure to below AP and markedly reduces PVA (double line).

Fig 3 shows representative PV loops at 3 levels of LVAD support in a normal dog.

p-LVAD slightly increased end-systolic pressure and volume, and decreased end-diastolic volume, and thus decreased stroke work modestly compared to control. In contrast, t-LVAD markedly decreased end-diastolic volume, shifting the PV loop leftward and yielding an extremely small PV loops.

**Fig 4:**
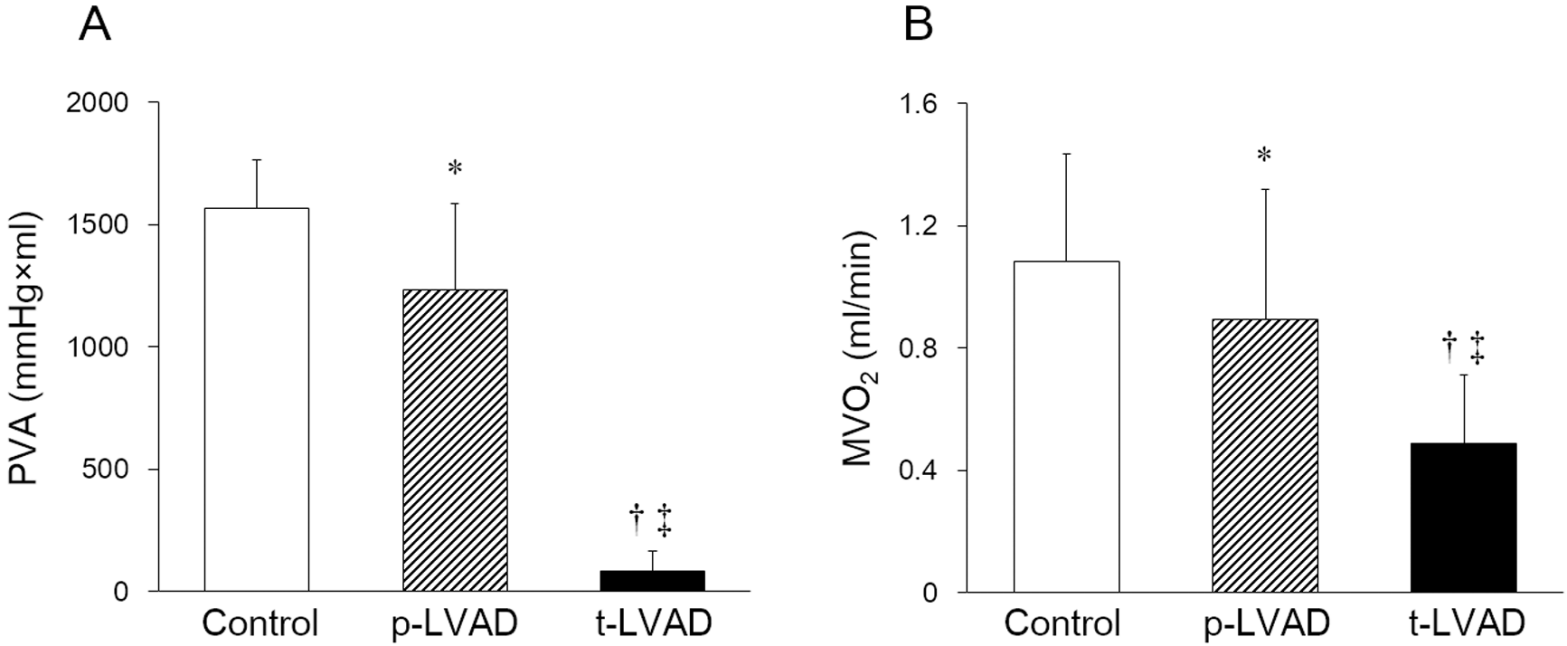
Impact of level of left ventricular assist device (LVAD) support on pressure-volume area and myocardial oxygen consumption in normal dogs. Open bar, hatched bar and closed bar indicate no LVAD support (Control), partial LVAD support (p-LVAD) and total LVAD support (t-LVAD), respectively. Data are expressed as means ± SD. p-LVAD marginally reduces PVA, while t-LVAD markedly decreases pressure-volume area (PVA). In addition, t-LVAD markedly reduces myocardial oxygen consumption (MVO_2_) by more than 55%. *p < 0.05 versus Control; ^†^*p* < 0.01 versus Control; ‡*p*<0.01 versus p-LVAD.

Fig 4 represents the impacts of LVAD on PVA and MVO_2_ in normal dogs. Compared to control, p-LVAD marginally reduced PVA, while t-LVAD markedly reduced PVA by more than 90% (Control: 1565 ± 200, p-LVAD: 1233 ± 353 and t-LVAD: 85 ± 81 mmHg×ml; *p*<0.05: p-LVAD vs. Control; *p*<0.01: t-LVAD vs. Control and p-LVAD). Furthermore, t-LVAD reduced MVO_2_ by 56±9% (Control: 1.08 ± 0.35, p-LVAD: 0.89 ± 0.43 and t-LVAD: 0.49 ± 0.22 ml/min; *p*<0.05: p-LVAD vs. Control; *p*<0.01: t-LVAD vs. Control and p-LVAD).

### Hemodynamics

The ischemia–reperfusion model was prepared in 18 dogs. Two dogs in control group died due to ventricular fibrillation within 90 min after LAD ligation and were excluded from analyses. Shown in Table 2 are hemodynamics before ischemia, 90 min after ischemia and 300 min after reperfusion. At baseline, mean AP and total CO (native CO) were not significantly different among the three groups. Following ischemia and reperfusion, both p-LVAD and t-LVAD significantly lowered LAP compared to control. t-LVAD lowered LV peak-systolic pressure to below mean AP resulting in zero CO at the LV, indicating the establishment of total LVAD-dependent circulation.

**Table 2:** Impact of level of left ventricular assist device (LVAD) support on hemodynamics in a dog model of ischemia-reperfusion.

	HR	MAP	CO	LVAD flow	RAP	LAP	Peak LVP
	(bpm)	(mmHg)	(ml/min/kg)	(ml/min/kg)	(mmHg)	(mmHg)	(mmHg)
Baseline							
Control	149±7	101±18	96±13	0	3.0±1.3	5.7±1.5	−
p-LVAD	144±17	96±11	92±10	0	3.0±0.7	5.3±0.8	−
t-LVAD	141±7	94±13	103±13	0	3.5±1.1	6.0±0.9	−
90 min after ischemia							
Control	132±18	98±19	106±23	0	4.2±1.9	9.9±2.9	−
p-LVAD	139±20	114±18	63±16*	65±18*	3.2±1.2	5.0±3.0*	−
t-LVAD	147±4	115±13	0*†	125±24*†	3.3±0.8	0.9±0.3*†	32±2
300 min after reperfusion							
Control	111±13	97±18	68±13	0	4.4±1.7	11.5±4.8	−
p-LVAD	117±11	99±12	26±4*	28±4*	3.0±2.0	7.9±4.5*	−
t-LVAD	124±11*	104±20	0*†	62±16*†	4.0±0.6	0.5±0.3*†	28±7

Data are expressed as means ± SD. Control, no LVAD support (n = 6); p-LVAD, partial LVAD support (n = 5); t-LVAD, total LVAD support (n = 5); HR, heart rate; MAP, mean arterial pressure; CO, cardiac output; LVAD flow, flow of left ventricular assist device; RAP, right atrial pressure; LAP, left atrial pressure; Peak LVP, peak LV pressure.

*p < 0.05 versus Control;

^†^*p* < 0.05 versus p-LVAD.

### Infarct size

**Fig 5:**
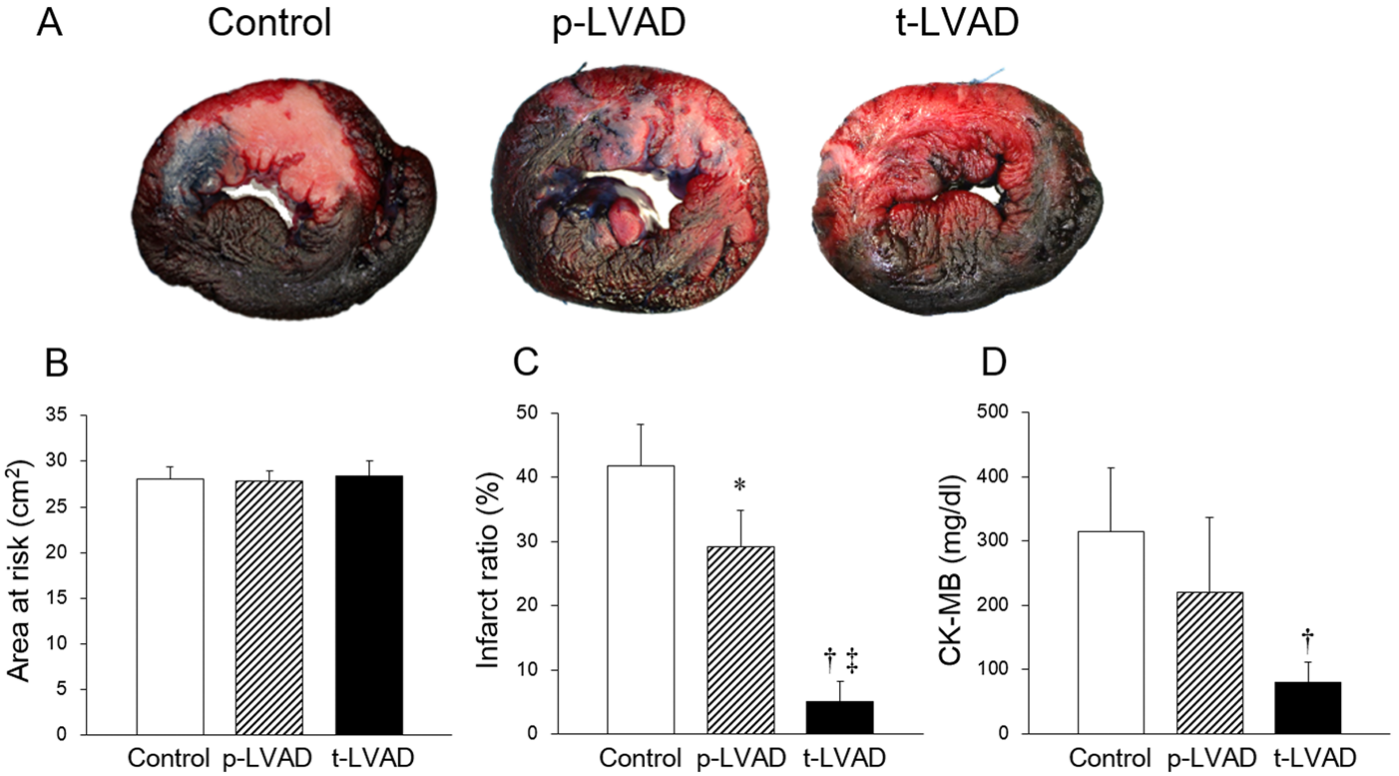
Impact of LVAD support level on infarct size in a dog model of ischemia reperfusion. (**A):** Representative left ventricular (LV) sections after staining with Evan’s blue and TTC. The area at risk (red) and the infarct area (white) were traced and measured by using an image analyzer. (**B):** Quantification of risk area in LV. There are no difference among three groups. (**C):** Assessment of infarct ratio (infarct area/risk area, %). Compared to control (no LVAD support, n = 6), p-LVAD (n = 5) moderately lowers the infarct ratio (*p* = 0.011), while t-LVAD (n = 5) strikingly decreases the infarct ratio by more than 80% (*p*<0.01). (**D)**: Changes in serum creatine kinase-MB (CK-MB) 300 min after reperfusion in the three groups. (**B**-**D)**: Data are expressed as means ± SD. *p < 0.05 versus Control; ^†^*p* < 0.01 versus Control; ‡*p*<0.01 versus p-LVAD.

We determined the infarct area and the area at risk by Evan’s blue stain and TTC stain, respectively (Fig 5A). The area at risk assessed by the manual tracing of red and white area did not differ among three groups (Control: 28.1 ± 3.0, p-LVAD, 27.8 ± 2.5 and t-LVAD: 28.4 ± 2.9 cm^2^; Fig 5B). As shown in Fig 5C, p-LVAD moderately lowered the infarct ratio (infarct area/area at risk), whereas t-LVAD strikingly decreased the infarct ratio by more than 80% (Control: 41.8 ± 6.4, p-LVAD: 29.1 ± 5.6 and t-LVAD: 5.0 ± 3.1%; *p* = 0.011: p-LVAD vs. Control; *p*<0.01: t-LVAD vs. Control and p-LVAD). The changes in CK-MB followed the same trend as infarct size (Control: 343.6 ± 77.6, p-LVAD: 220.3 ± 116.4 and t-LVAD: 79.6 ± 32.2 mg/dl; *p* = 0.11: p-LVAD vs. Control; *p*<0.01: t-LVAD vs. Control, Fig 5D).

## Discussion

We investigated the impact of LVAD on LV work and infarct size in a canine model of ischemia-reperfusion. The major findings of this study are as follows: (1) Partial LVAD support did not markedly reduce PVA or oxygen consumption of LV. On the other hand, total LVAD support strikingly reduced LVV, PVA and oxygen consumption of LV. (2) Total LVAD support markedly reduced the infarct size in a canine model of ischemia-reperfusion.

### Effect of LVAD on PVA and MVO_2_

In the era of widespread use of LVAD, the understanding of mechanical and physiological impact of LVAD on LV is critically important for maximizing the benefits while minimizing the risks associated with the management of LVAD. As we hypothesized (Fig 1), p-LVAD moderately decreased PVA and LV MVO_2_, while t-LVAD markedly reduced both (Figs 3 and 4). This is because p-LVAD decreases LV end-diastolic volume but increases net total cardiac output (CO + LVAD flow), which in turn increases AP and end-systolic volume of LV. As a result, PVA does not decrease markedly despite the reduction in LV stroke volume. In this setting, the mechanical effect of LVAD on LV is similar to extracorporeal membrane oxygenation [14]. On the contrary, t-LVAD decreases end-systolic volume as well as end-diastolic volume of LV, because LV no longer ejects and LV pressure is totally dissociated from AP. Thus, t-LVAD markedly reduces PVA by more than 90% and nearly halved MVO_2_. Suga et al. [10] demonstrated a tight linear correlation between PVA and LV MVO_2_ in normal dogs, and showed that the PVA independent MVO2 (i.e, the MVO_2_-axis intercept of the MVO_2_-PVA regression line) reflects the LV basal metabolism including the energy required for excitation-contraction coupling. Our data are consistent with their results, and the residual MVO_2_ during t-LVAD (44 ± 9%) may account for such PVA-independent energy utilization by the LV.

Several studies have addressed the impact of LVAD support on LV work and MVO_2_. Meyns et al. [15] reported the impact of different levels of LV unloading with a transvascular LVAD (Impella^®^) on MVO_2_ in sheep. Stratification of LVAD support level in their study differed from ours. They defined full LVAD support as LVAD flow at maximal rotational speed and partial LVAD support as half of the baseline CO provided by LVAD. In their study, even with full LVAD support, the native LV remained ejecting. Thus, LVAD failed to totally unload LV in their full LVAD support group and reduced MVO_2_ by no more than 30%. In addition, Morley et al. [16] reported the impact of partial LVAD support on the PV loop in a simulation study. Their simulated partial LVAD support was almost the same as ours, suggesting no marked reduction of PVA.

**Fig 6:**
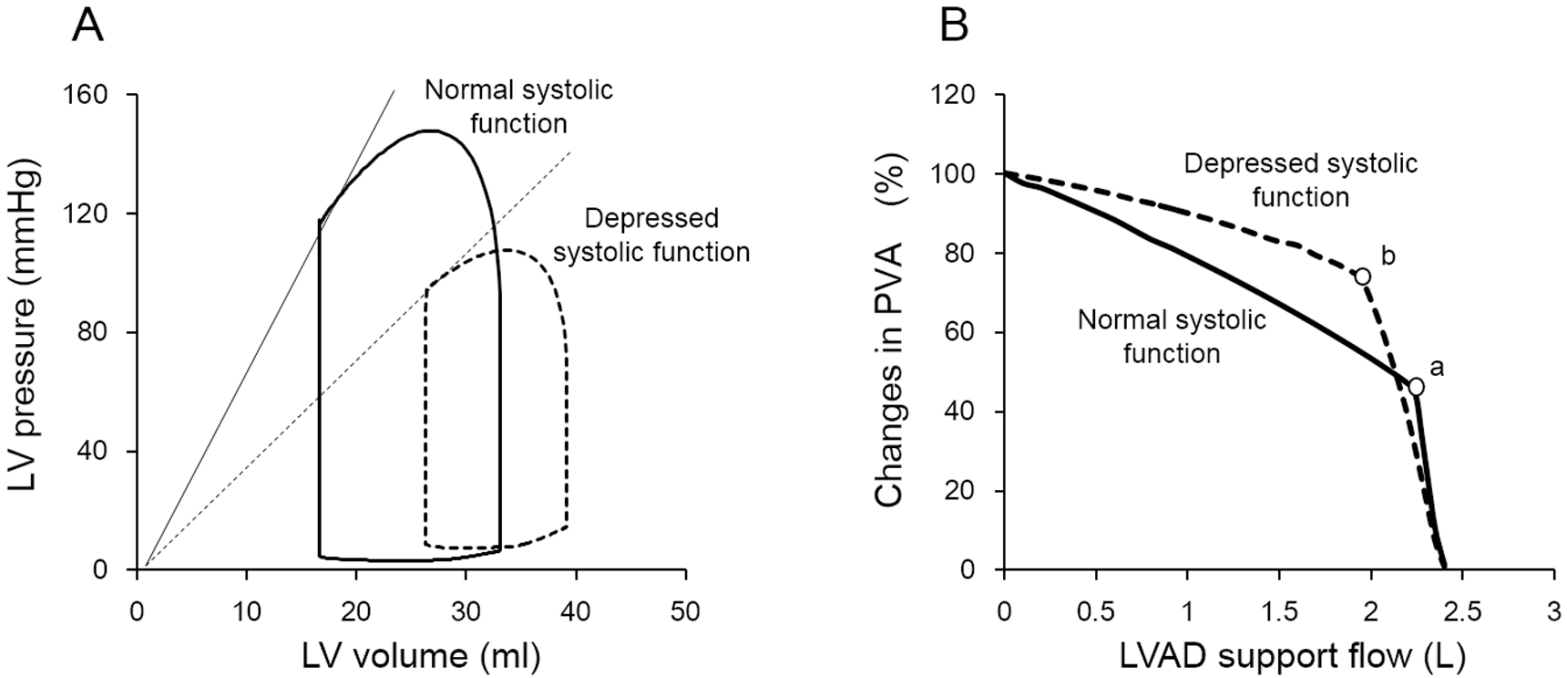
Simulation study with normal and depressed LV systolic function. (**A)**: Simulated PV loop of LV with normal (solid line, maximum elastance: 7 mmHg/ml) and depressed (dashed line, maximum elastance: 1 mmHg/ml) systolic function. Mean circulatory filling pressure was fixed in both systolic conditions (6.4 mmHg). **(B)**: The relationship between LVAD support flow and changes in PVA in normal (solid line) and depressed (dashed line) LV systolic function. PVA at baseline (no LVAD support) were normalized at 100% in each group. Both “a” and “b” indicate the inflection point which circulation turns to total LVAD from partial LVAD support. The rate of decrease in PVA by partial LVAD support (baseline PVA to that of “a” and “b”) is much lower in depressed LV (-27%) than in normal LV (-54%).

It is conceivable that the impact of LVAD unloading on PVA and MVO2 may differ depending on the basal contractibility of LV. To examine this possibility, we examined the impact of LVAD support on PVA in silico using the 10-element cardiovascular model (S1 Fig). We developed the dynamic cardiovascular system by using Simulink (Mathworks, Massachusetts, USA). Both the systemic and pulmonary circulation are modeled by using 5-elemennt resistance-capacitance net-work model. We approximated 4 intracardiac valves as a unidirectional valve. Four cardiac chambers are represented valves as a unidirectional valve. Four cardiac chambers are represented by time-varying elastance [17, 18]. LVAD was designed to withdraw blood from LV and returned it to aorta continuously. Parameter values were shown in S1 Table. As shown in Fig 6, under both normal and depressed LV contraction, increasing partial LVAD support flow marginally decreases PVA, while total LVAD support markedly reduces PVA. However, the rate of decrease in PVA by partial LVAD support is much lower in the depressed LV (-27%) than in the normal LV (-54%). Hence, partial LVAD does not reduce PVA substantially, especially in the depressed LV. This is because LVAD induces net increases in cardiac output, and AP impedes ejection in the depressed LV and significantly increases potential energy (PE) of PVA [19, 20]. Therefore, total LVAD unloading is prerequisite to effectively unload LV with poor LV systolic function such as the heart with myocardial infarction.

### Effect of total LVAD support on infarct size

Although the wide-spread use of early coronary reperfusion therapy successfully provides rapid restoration of oxygen supply for the ischemic myocardium, earlier initiation of reperfusion remains a challenge in clinical settings. Meanwhile, several treatments such as beta blockers [21, 22] and ACE inhibitors [23, 24] that reduce MVO_2_ have been proposed to reduce infarct size in animal models of MI or ischemia-reperfusion. Since these drugs reduce AP, HR and cardiac contractility, there is a risk that they may destabilize hemodynamics in acute MI. As shown in Table 2, t-LVAD during ischemia-reperfusion succeeded to reduce LAP while maintaining CO and AP, indicating that t-LVAD unloads LV and reduces MVO_2_ without having an adverse effect on hemodynamics.

In this study, we showed that p-LVAD moderately reduced infarct size, while t-LVAD markedly reduced infarct size by more than 80% (Fig 5). Since an imbalance between oxygen supply and demand is the fundamental underlying pathophysiology that leads to the formation of infarcted myocardium, we conjecture that reduction of MVO2 during ischemia is essential in decreasing infarct size in this study. However, there are other possible explanations, which may not be mutually exclusive, why LVAD unloading reduces infarct size. With regard to ischemia-reperfusion injury, Kapur et al. [25] reported that LVAD unloading activates the RISK pathway, that protectes the myocardium from apoptosis. Tamareille et al. [26] also revealed that LVAD unloading before reperfusion reduces the release of endothelin-1 and inhibits calcium overload and apoptosis. Flameng et al. [27] indicated the importance of collateral blood flow in a dog model of MI. Since collateral blood flow is elevated by increased arterial diastolic pressure and decreased LV end-diastolic pressure [28, 29], the improvement of collateral flow by LVAD may contribute to the reduction of infarct size. Further investigation is needed to clarify how much these factors are different between p-LVAD and t-LVAD. In addition, as shown in these previous studies, the finding that LVAD unloading reduced the infarct size in MI heart was not surprising. What we want to emphasize in this study is that the level of mechanical unloading differs greatly different between p-LVAD and t-LAVD, and that t-LVAD has much greater anti-infarct effect.

Reduction of infarct size may result in suppression of cardiac remodeling in the long term. Thus, the development of total LAVD support as an acute medical treatment in MI may be expected to reduce long-term heart failure. We also need to investigate the impact of t-LVAD therapy on long-term cardiac remodeling and heart failure after MI.

Currently, transvascular LVAD systems such as Impella^®^ and TandemHeart^™^ allow unloading of the LV in acute clinical setting of MI. The question of when is the optimal timing of LVAD initiation is of great importance. Kapur et al. [25] proposed a novel strategy which they named “Door to Unload” and demonstrated that earlier unloading is better than earlier reperfusion in reducing infarct size. On the other hand, Achour et al. [30] reported that LVAD unloading after reperfusion did not reduce infarct size in a dog model of ischemia reperfusion. In this study, we started treatment at the beginning of ischemia to examine the maximum effect of t-LVAD. To apply the results of this investigation to clinical settings, we need to investigate the effect of starting t-LAVD after the onset of ischemia.

### Limitations

This study has several limitations. First, we cannot manage a collateral flow in the canine model. In dogs, the collateral circulation is known to contribute significantly to cell viability in both permanent occlusion and ischemia-reperfusion [27]. In addition, the degree of collateral circulation varies among individuals. Since we were not able to study collateral circulation in this study, we have to be very careful to extrapolate these results directly to acute MI patients. Nevertheless, the fundamental mechanism of myocardial infract is the imbalance between the supply and demand cannot be violated. Thus, the qualitative difference in the impact between t-LVAD and p-LVAD on infarct size is likely to remain in patients.

Second, evaluating the correlation between PVA (or MVO2) reduction and infarct size is important to better understand how LVAD impacts infract size. However, we did not measure PVA or MVO2 in animal models of ischemia-reperfusion injury because of technical difficulties such as LV volume measurement using sonometric volumetry and coronary blood flow measurement in the LAD-occluded ischemic heart. Instead, we examined the impact of LVAD support level on PVA and MVO2 only in normal dogs to proof our concept. As shown in the simulation study (Fig 6), LVAD markedly reduces PVA irrespective of LV function although the reduction depends on the level of LVAD support (p-LVAD and t-LVAD). Therefore we speculate that the general trends of how LVAD modulates PVA and MVO2 remain valid in canine ischemia-reperfusion models.

Third, the LVAD we used in this study was not the same as the device used clinically. Since our study is based on the concept of mechanical unloading and LV oxygen consumption, the mechanical effect of the LVAD on the left heart is not different between our device and a clinical device. Thus, the difference in device would not have affected our results.

Fourth, a pump that can be inserted percutaneously and support over 5 L/min is currently unavailable. In humans, pumping capacity of at least 5 L/min is required to maintain total LVAD-dependent circulation. To develop the proposed t-LVAD as a clinical option, development of high flow percutaneous pumps is a prerequisite.

Fifth, we opened the chest and removed the pericardium in this experiment. This procedure would have decompressed the heart, decreased biventricular interaction and pericardial constraint, and reduced LVEDP, consequently reducing the infarct size. Thus, we cannot exclude the possibility that chest opening and pericardiectomy attenuated the favorable impact of LVAD unloading on reducing infarct size, because these procedures per se could reduce infarct size in myocardial infarction.

Lastly, in this investigation, we started the LVAD support from the beginning of ischemia which is very unlikely in real clinical settings. Since previous reports indicated that earlier LVAD unloading prior to reperfusion is better to reduce infarct size [25, 30], we hypothesized that delaying the timing of LVAD unloading may attenuate the impact of LVAD on infarct size. However, as we discussed above, the purpose of this study was to demonstrate the difference between p-LVAD and t-LVAD in reducing the infarct size. We have shown that t-LVAD is by far better than p-LVAD in reducing the MI size. How delaying the initiation of LVAD unloading affects the differential impact between p-LVAD and t-LVAD on the MI size remains to be investigated.

## Conclusions

In conclusion, total LVAD support greatly reduced the left ventricular pressure-volume area, and thus suppressed left ventricular oxygen consumption. In a canine model of ischemia-reperfusion, total LVAD support markedly improved hemodynamics and reduced infarct size. Total LVAD may serve as a powerful option in the treatment of acute myocardial infarction.

## Supporting Information

S1 Fig The 10-elements circulatory model. ELV, time varying elastance of left ventricle; CSA, compliance of systemic artery; CSC, compliance of systemic capillary vessels; CSV, compliance of systemic vein; ERA, time varying elastance of right atrium; ERV, time varying elastance of right ventricle; CPA, compliance of pulmonary artery; CPC, compliance of pulmonary capillary vessels; CPV, compliance of pulmonary vein; ELA, time varying elastance of left atrium; DAV, Aortic valve; DTV, Tricuspid valve; DPV, Pulmonary valve; DMV, Mitral valve; RAV, resistance of aortic valve; RA, resistance of aorta; RSA, resistance of systemic artery; RSC, resistance of systemic capillary vessels; RSV, resistance of systemic vein; RTV, resistance of tricuspid valve; RPV, resistance of pulmonary valve; RPA, resistance of pulmonary artery; RPC, resistance of pulmonary capillary vessels; RPV, resistance of pulmonary vein; RMV, resistance of mitral valve.
(TIF)

S1 Table Parameter values characterizing the cardiovascular system. CSA, compliance of systemic artery; CSC, compliance of systemic capillary vessels; CSV, compliance of systemic vein; CPA, compliance of pulmonary artery; CPC, compliance of pulmonary capillary vessels; CPV, compliance of pulmonary vein; RAV, resistance of aortic valve; RA, resistance of aorta; RSA, resistance of systemic artery; RSC, resistance of systemic capillary vessels; RSV, resistance of systemic vein; RTV, resistance of tricuspid valve; RPV, resistance of pulmonary valve; RPA, resistance of pulmonary artery; RPC, resistance of pulmonary capillary vessels; RPV, resistance of pulmonary vein; RMV, resistance of mitral valve.
(DOCX)

## Abbreviations

AP: arterial pressure
CO: Cardiac output
EDPVR: end-diastolic pressure-volume relationship
ESPVR: end-systolic pressure-volume relationship
HR: heart rate
LAD: left anterior descending coronary artery
LAP: left atrial pressure
LV: left ventricle
LVAD: left ventricular assist device
LVV: left ventricular volume
MI: acute myocardial infarction
MVO2: myocardial oxygen consumption
PVA: pressure-volume area
RAP: right atrial pressure
