# Supplementary figures and images for: Total Mechanical Unloading Minimizes Metabolic Demand of Left Ventricle and Dramatically Reduces Infarct Size in Myocardial Infarction
Source: PLoS One. 2016 Apr 28;11(4):e0152911. doi: 10.1371/journal.pone.0152911 (PMC4849631; doi:10.1371/journal.pone.0152911)

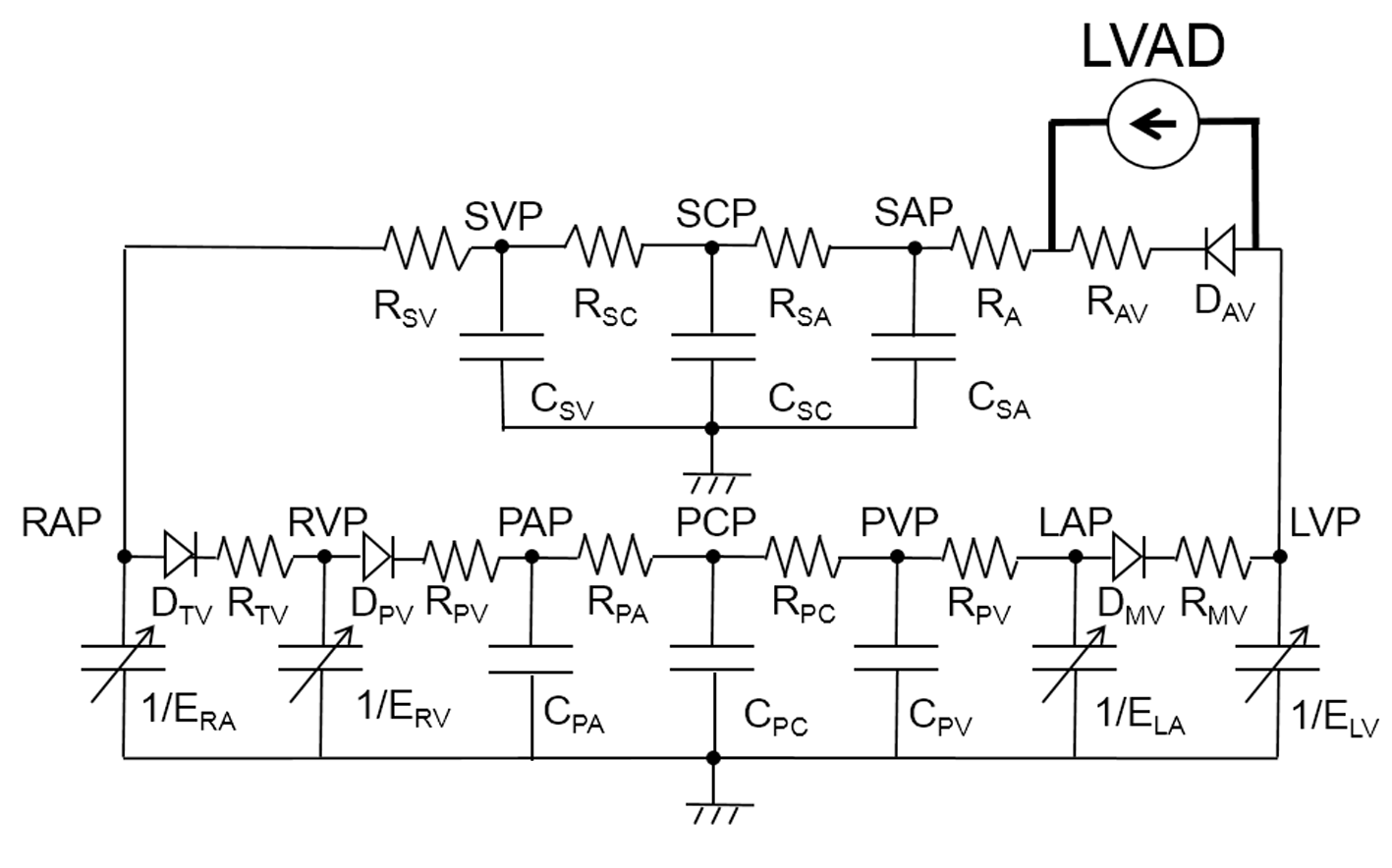

Supplement: S1 Fig — ELV, time varying elastance of left ventricle; CSA, compliance of systemic artery; CSC, compliance of systemic capillary vessels; CSV, compliance of systemic vein; ERA, time varying elastance of right atrium; ERV, time varying elastance of right ventricle; CPA, compliance of pulmonary artery; CPC, compliance of pulmonary capillary vessels; CPV, compliance of pulmonary vein; ELA, time varying elastance of left atrium; DAV, Aortic valve; DTV, Tricuspid valve; DPV, Pulmonary valve; DMV, Mitral valve; RAV, resistance of aortic valve; RA, resistance of aorta; RSA, resistance of systemic artery; RSC, resistance of systemic capillary vessels; RSV, resistance of systemic vein; RTV, resistance of tricuspid valve; RPV, resistance of pulmonary valve; RPA, resistance of pulmonary artery; RPC, resistance of pulmonary capillary vessels; RPV, resistance of pulmonary vein; RMV, resistance of mitral valve. (TIF) [file pone.0152911.s001.tif]
