# Supplementary material for: Total Mechanical Unloading Minimizes Metabolic Demand of Left Ventricle and Dramatically Reduces Infarct Size in Myocardial Infarction
Source: PLoS One. 2016 Apr 28;11(4):e0152911. doi: 10.1371/journal.pone.0152911 (PMC4849631; doi:10.1371/journal.pone.0152911)
Supplement: S1 Table — CSA, compliance of systemic artery; CSC, compliance of systemic capillary vessels; CSV, compliance of systemic vein; CPA, compliance of pulmonary artery; CPC, compliance of pulmonary capillary vessels; CPV, compliance of pulmonary vein; RAV, resistance of aortic valve; RA, resistance of aorta; RSA, resistance of systemic artery; RSC, resistance of systemic capillary vessels; RSV, resistance of systemic vein; RTV, resistance of tricuspid valve; RPV, resistance of pulmonary valve; RPA, resistance of pulmonary artery; RPC, resistance of pulmonary capillary vessels; RPV, resistance of pulmonary vein; RMV, resistance of mitral valve. (DOCX) [file pone.0152911.s002.docx]

**Supporting information**

| Compliance  (ml/mmHg) | | Resistance  (mmHg/ml/sec) | |
| --- | --- | --- | --- |
| C_SA_ | 0.4 | R_AV_ | 0.05 |
| C_SC_ | 40 | R_A_ | 0.2 |
| C_SV_ | 12 | R_SA_ | 3 |
| C_PA_ | 2.4 | R_SC_ | 0.1 |
| C_PC_ | 8 | R_SV_ | 0.1 |
| C_PV_ | 2 | R_TV_ | 0.005 |
|  |  | R_PV_ | 0.03 |
|  |  | R_PA_ | 0.2 |
|  |  | R_PC_ | 0.05 |
|  |  | R_PV_ | 0.03 |
|  |  | R_MV_ | 0.005 |

**S1 Table. Parameter values characterizing the cardiovascular system.**
